# Analysis of SF-6D Health State Utility Scores: Is Beta Regression Appropriate?

**DOI:** 10.3390/healthcare8040525

**Published:** 2020-12-01

**Authors:** Samer A Kharroubi

**Affiliations:** Department of Nutrition and Food Sciences, Faculty of Agricultural and Food Sciences, American University of Beirut, Beirut 1107-2020, Lebanon; sk157@aub.edu.lb; Tel.: +961-1-350-000 (ext. 4541)

**Keywords:** beta regression, preference-based health measure, SF-6D system, MCMC, Bayesian methods

## Abstract

*Background*: Typically, modeling of health-related quality of life data is often troublesome since its distribution is positively or negatively skewed, spikes at zero or one, bounded and heteroscedasticity. *Objectives*: In the present paper, we aim to investigate whether Bayesian beta regression is appropriate for analyzing the SF-6D health state utility scores and respondent characteristics. *Methods*: A sample of 126 Lebanese members from the American University of Beirut valued 49 health states defined by the SF-6D using the standard gamble technique. Three different models were fitted for SF-6D via Bayesian Markov chain Monte Carlo (MCMC) simulation methods. These comprised a beta regression, random effects and random effects with covariates. Results from applying the three Bayesian beta regression models were reported and compared based on their predictive ability to previously used linear regression models, using mean prediction error (MPE), root mean squared error (RMSE) and deviance information criterion (DIC). *Results*: For the three different approaches, the beta regression model was found to perform better than the normal regression model under all criteria used. The beta regression with random effects model performs best, with MPE (0.084), RMSE (0.058) and DIC (−1621). Compared to the traditionally linear regression model, the beta regression provided better predictions of observed values in the entire learning sample and in an out-of-sample validation. *Conclusions*: Beta regression provides a flexible approach to modeling health state values. It also accounted for the boundedness and heteroscedasticity of the SF-6D index scores. Further research is encouraged.

## 1. Introduction

A large number of preference-based measures of health-related quality of life (HRQoL) have been used to measure quality-adjusted life years (QALYs) to be applied in cost-effectiveness analyses (CEA). Such measures comprise the EQ-5D [1], HUI2 and 3 [2,3], AQoL [4], QWB [5] and the SF-6D [6], along with several condition-specific preference-based measures [7,8]. A key practical issue with these measures is that they produce many distinct health states, and so it may not be practical to get a direct valuation for every health state. Toward that end, statistical models are then needed to estimate utilities for all states produced by the measure based on the valuations of a representative sample of health states.

Typically, the distribution of health state utilities obtained from valuation techniques like time tradeoff (TTO) or standard gamble (SG) is positively or negatively skewed, truncated, hierarchal as well as noncontinuous, all of which impose a key challenge for statistical modeling methods. Despite this challenge, earlier statistical methods of such data have used ordinary least-squares regression (OLS) [6,9,10]. However, the error terms in the OLS regression are assumed to be homoscedastic, so such assumption may not be met for bounded variables as the variance of scores decreases when the mean gets closer to the boundaries [11,12]. An additional assumption associated with the error terms is that they are normally distributed, and again this assumption may not hold [12].

A variety of alternative regression models available in the literature have been used to analyze HRQoL data (for example, Tobit models [13,14,15], latent class models (LCM) [14] and two-part models (TPM) [14,16]). Although Tobit models are defined to allow for censoring, they may not be appropriate for HRQoL data because scores are measured on a scale where values cannot go beyond one [12]. On the other side, LCM and TPM models have shown good properties in handling ceiling effects like that of the EQ-5D [14,16]. However, they may be less relevant for modeling SF-6D data where the proportion of values at one was low [16,17].

Beta regression, proposed by Ferrari and Cribari-Neto [18], provides a flexible approach to analyzing HRQoL data as it allows modeling positively or negatively skewed (and heteroscedastic) distributed outcomes. A number of published research have focused on analyzing HRQoL observations using beta regression models. See Hubben et al. [19] and Moberg et al. [20], who used beta regression for analyzing EQ-5D and visual analog scale datasets, respectively. Basu and Manca [21] found that beta regression performed better than conventional OLS methods upon regressing HRQoL outcomes on covariates. Cheung et al. [22] used beta regression with dispersion covariates to model SF-36 index data. Further, Hunger et al. [23] have also used beta regression to model the association between respondent characteristics and SF-6D data.

The aim of this paper is to develop alternative regression methods based on beta distribution to tackle the aforementioned characteristics of the HRQoL data. More specifically, we aim to investigate whether Bayesian beta regression is appropriate for analyzing the SF-6D health state utility scores and respondent characteristics. While a wider range of regression models have been proposed to analyze HRQoL data [13,14,15,16], estimated effects and predictive ability of the proposed models are compared against the traditionally used linear regression model.

The Section 2 of the paper briefly presents the SF-6D Lebanon valuation study, along with the data collection procedure. Full details of the valuation study and the data collection process are available elsewhere [24]. The models used for the data analysis, including model estimation, reliability and validation, are also presented. The findings obtained from every method while comparing the models in terms of their estimated effects and predictive accuracy are set out in the Section 3. The Section 4 finishes off with a general discussion of the findings along with some limitations for the study and possible directions for further research.

## 2. Materials and Methods

### 2.1. The SF-6D

The SF-6D is a generic measure of health states attained from the SF-36. This preference-based measure includes 6 dimensions: physical functioning, role limitations, social functioning, pain, mental health, and vitality. Every dimension has between 4 and 6 levels [6]. Health states of the SF-6D are represented by a six-digit number through choosing a level from every dimension, beginning with physical functioning and finishing off with vitality, generating 18,000 possible combinations in total. The best health state is expressed as 111111, which is the perfect health state indicating no loss of health in any dimension, while the descriptor 645,655 represents the worst health state known as “the pits”.

### 2.2. Study Design

A sample of 126 Lebanese respondents from AUB valued 49 health states described through the SF-6D using the SG technique. Selection of respondents, along with a selection of health states, have been reported elsewhere [24].

#### 2.2.1. Selection of Respondents

Lebanese adults above 18 years old were recruited at AUB and were stratified via sex (female/male) and profession (faculty/staff/students). Respondents were approached by telephone and/or email to arrange for a one-to-one interview session. Out of the 170 respondents who were initially contacted, a total of 126 people agreed to participate in the study and conducted the interview session (response rate = 74%). Reasons for declining to participate were mainly due to time constraints and disinterest in the study. More on the selection method is given in Kharroubi et al. [24].

#### 2.2.2. Selection of Health States

The sample of health states was chosen using the Orthoplan procedure of SPSS (SPSS Inc., Chicago, IL, USA) that generates 49 out of the 18,000 possible states to fit an additive model. The 49 health states were then distributed over 7 sets with 7 health states in every set. Additionally, all respondents valued the “pits” state. More details are presented in Kharroubi et al. [24].

#### 2.2.3. Interviews

During the interview, the respondent was asked to rank and then value eight SF-6D health states via the McMaster “ping pong” variant of SG, where every respondent is asked to value each of the seven certain health states versus the best state and the “pits” [25]. As for the eighth SG question, the respondent was asked to value the “pits” state. Based on their valuation of the “pits”, each respondent was asked to choose between either: (A) the certain prospect of being in the “pits” and the uncertain prospect of best health or immediate death; or (B) the certain prospect of death and the uncertain prospect of best health or the “pits”. The chances that the best outcome occurring are varied until the respondent is indifferent between the two prospects. Any negative value was bounded to −1, and it referred to worse than death [26]. The seven earlier health state valuations (SG) were adjusted onto the scale 1 to 0, with 1 being the perfect health state and 0 indicating death [6], via the following formula:

SGADJ = SG + (1 − SG) × P

where P is the valuation of the “pits”. The resulting SGADJ values represent the response variable (*y*) in the models described below.

### 2.3. Study Sample

As already mentioned, each respondent was asked to value eight SF-6D states, including “pits”. This results in 1008 health state valuations across 49 health states. There were two respondents whose valuations did not change between the eight health states, and so they had to be excluded from the analysis, leaving in total 992 (98.4%) SG valuations across 49 health states for the analysis. Every health state was valued 17 or 18 times, with average values ranged from 0.322 (pits state) to 0.890 (state 211,111) and standard deviations ranged from 0.042 (211,111) to 0.265 (614,434). However, the median values were higher, indicating the left skewness of the data. Negative values did not occur, and over 19% of observations were above 0.9. Finally, no states were evaluated at 1, indicating that all respondents were inclined to risk a worse state to get a chance for a better one. More details on health states, values are available in Kharroubi et al. [24].

### 2.4. Modeling

#### 2.4.1. Model Development

As already mentioned, all studies that modeled SF-6D index scores as a function of respondent characteristics relied on linear regression using OLS [6,9,10]. However, linear regression assumes that error terms are normally distributed with constant variance (homoscedastic), both of which may not be met for bounded variables. Here, we develop alternative regression methods based on beta distribution to analyze the SF-6D health state utility scores and respondent characteristics.

Three different models were fitted for the SF-6D via Bayesian Markov chain Monte Carlo (MCMC) simulation methods. These comprised a beta regression, random effects and random effects with covariates. For every model, the SF-6D index score was used as the dependent variable. The dummy explanatory variables for each level above 1 from each of the six dimensions of the SF-6D were all used as independent variables.

##### Beta Regression

The beta distribution is a natural choice for modeling continuous variables observed in the standard unit interval. The probability density function of a random variable *y* following a beta distribution is defined as:

(1) $$f\left(y|\mu ,\phi \right)=\frac{\Gamma \left(\phi \right)}{\Gamma \left(\mu \phi \right)\Gamma \left(\left(1-\mu \right)\phi \right)}y^{\mu \phi -1}{\left(1-y\right)}^{\left(1-\mu \right)\phi -1},\;0<y<1$$

where Γ(.) denotes the gamma function [27]. Note that μ (0<μ<1) represents the expected value of *y*. The parameter ϕ denotes a precision parameter since μ=E(y) and Var(y)=μ (1 − μ)/(1 + ϕ) and, hence, for fixed μ, 1 + ϕ would be inversely proportional to Var(y). Hence, if *y* has the probability density function (1), we write

y ~beta(μϕ,(1−μ)ϕ)

Now, assume $y_{1},\ldots ,\;y_{n}$ to be *n* independent and identically distributed random variables such that $y_{i}\;\sim \mathrm{beta}\left(\mu_{i}\phi ,(1-\mu_{i}\right)\phi ),i=1,\;\ldots ,\;n$. By definition, the mean $\mu_{i}=E\left(y_{i}\right)$ maps the unit interval onto the real line, so the logit link was proposed as a convenient link function. That is,

(2) $$\log\frac{\mu_{i}}{1-\mu_{i}}=x_{i}^{T}\beta ,$$

where $x_{i}^{T}$ denotes a vector of covariates for the *i*-th subject (i.e., the 25 dummy explanatory variables described above), and β denotes a vector of regression parameters.

The beta regression (BR) model outlined above could also involve random effects that account for variation within and between respondents. This type of model recognizes that *n* observations from *m* individuals are not treated as *n* × *m* observations on different respondents. The logit link function (2) is now:

(3) $$\log\frac{\mu_{i}}{1-\mu_{i}}=x_{i}^{T}\beta +b_{i},$$

where

$$b_{i}\sim N\left(0,\sigma_{1}^{2}\right)$$

Note that $b_{i}$ indicates the “*i*-th respondent” random effect for the HRQoL utility value. This allows many respondents to have consistently low or high average HRQoL utility scores. Note also that $\mathrm{Var}\left(b_{i}\right)=\sigma_{1}^{2}$, *i* = 1,…, *n*, represents the variability in the average HRQoL among respondents with comparable characteristics.

The BR model could also involve participant characteristics, for example, age, gender, marital status, education level, housing type and income, with the aim of capturing the impact of the participant covariates.

The Bayesian beta regression model is finalized by assigning prior distributions for all unknown parameters. In the case when no specific prior information exists, it would be possible to assign non-informative prior distributions for all these parameters. Typically, multivariate normal prior distributions are assigned for the fixed effects with mean equal to zero and with large variance. A common choice for the prior distributions of the variance of random effects, $\sigma_{1}^{2}$, and the precision parameter ϕ would be an inverse gamma distribution. More specifically, prior distributions were specified as follows:

$$\beta \sim N\left(0,10^{6}\right),\;\sigma_{1}^{2},\phi \sim IG\left(0.001,0.001\right)$$

Gelman [28] suggests a flat prior for σ, hence the prior for $\sigma_{1}^{2}$ is proportional to $\sigma^{-1}$. Gelman also suggests that the prior distribution for $\phi =U^{2}$, where U~U(0,a) with big *a* (*a* = 50 say) is less informative than an inverse gamma prior distribution.

##### Linear Regression

The linear regression (LR) model is specified as follows:

(4) $$y_{i}=\;x_{i}^{T}\beta +\varepsilon_{i}$$

where $y_{i}$ is the SF-6D utility score of respondent *i* and $\varepsilon_{i}$ are random error terms with $E(\varepsilon_{i})=0$ and $Var(\varepsilon_{i})=\sigma_{2}^{2}$ for all *i*. Note here that the error terms are homoscedastic given they have constant variance for any *i*; however, they could be relaxed to take on non-constant variance (heteroscedasticity). The LR model could also involve random effects in addition to the participant characteristics (for example, age, gender, marital status, education level, housing type and income) to estimate the impact of the participant covariates. Vague prior distributions were defined as follows:

$$\beta \sim N\left(0,10^{6}\right),\;\sigma_{2}^{2}\sim IG\left(0.001,0.001\right)$$

See Natarajan and Kass [29] for more choices of non-informative prior distributions.

#### 2.4.2. Model Estimation

All models described above were evaluated via Gibbs sampling MCMC simulation methods and were implemented in the WinBUGS software package [30,31]. This environment permits great flexibility in model specification, which allows nonstandard models to be fitted in a straightforward manner. The relevant WinBUGS code is available from the author upon request. Following some initial test runs, it was decided that a burn-in of 5000 runs would be sufficient to reach convergence, and these runs were discarded from the final analysis. Convergence was examined using the Gelman and Rubin diagnostic [32]. Two parallel chains were set from broadly spread initial values, and the ratio of the within-chain to between-chain variance was then monitored and converged to about 1, indicating convergence had been reached. Following this, further 10,000 runs were executed for parameters estimation purposes.

#### 2.4.3. Model Reliability and Validation

The proposed models were compared in terms of their predictive performance via mean prediction error (MPE), root-mean-square error (RMSE) and the Bayesian deviance information criterion (DIC) [30]. The DIC is defined by:

$$DIC=\bar{D}+P_{D}$$

where $\bar{D}$ is the posterior mean deviance and $P_{D}$ is the effective number of parameters that represents model complexity. The model with the lowest DIC is preferred as the best fitting model [31].

## 3. Results

### 3.1. All Models

The posterior means and their associated 95% credible intervals (CI) for each of the regression coefficients are reported in Table 1. Notice that the estimates shown in bold are those who had CIs, excluding zero. For the LR model, we see that all regression coefficients had the expected negative sign apart from the second and third levels of vitality as well as the third level of role limitation. This is also the case for the BR model with one addition, level 2 of mental health. However, the CI of these coefficients for both models includes zero, which means that it is a weak inconsistency. We also see that 16 out of the 25 coefficients on the main SF-6D domains have CIs excluding zero in the LR model, in comparison to 15 coefficients for the BR model. The MPE, RMSE and DIC for each model are also reported in Table 1. We notice that the BR model scored better MPE, RMSE and DIC with 0.128, 0.049 and −1069, respectively, in comparison to the LR model (MPE = 0.126, RMSE = 0.053, DIC = −689.1).

Upon including random effects, we see that all regression coefficients were negative across both LR + RE and BR + RE models, except for level 2 of vitality and level 3 of pain. In addition, the 95% CI for these coefficients included zero in both models. Further, 16 out of the 25 coefficients on the main SF6D domains have CIs excluding zero in LR + RE model, in comparison to 15 coefficients for BR + RE model. With regard to the models’ performance, the BR + RE model showed better MPE, RMSE and DIC than the LR + RE model (LR + RE: MPE = 0.089, RMSE = 0.064, DIC = 1325; BR + RE: MPE = 0.084, RMSE = 0.058, DIC = 1621). Finally, to estimate the impact of the respondent characteristics, respondents’ age, sex, marital status, education level, housing type and income were added to both LR + RE and BR + RE models. The findings indicate that the 95% CI for these covariates coefficients contained zero in both models. However, the beta regression model showed better MPE, RMSE and DIC than the linear regression model (LR + RE + COV: MPE = 0.089, RMSE = 0.116, DIC = 1306; BR + RE + COV: MPE = 0.084, RMSE = 0.112, DIC = 1605).

Overall, the beta regression model was found to perform better than the normal regression model across all types of models, with/without random effects and with/without covariates. Given including covariates for both LR + RE and BR + RE models did not provide any further improvement in terms of MPE, RMSE and DIC, let alone the 95% CI for covariates coefficients contained zero in both models, we carry on our analysis with model BR + RE as the best performing model in the study. The estimates are compared to the LR + RE model using the different prediction criteria described above.

### 3.2. BR + RE vs. LR + RE

Table 2 shows the inference for the mean health state valuations of the 49 health states used in the sample. For every state, Table 2 displays the posterior mean and standard deviation from both LR + RE and BR + RE models, along with the actual mean utility. Across the 49 states that were used in the study, the predictive performance of BR + RE is generally better than that of LR + RE, with MPE and RMSE of 0.023 and 0.053, respectively, for the BR + RE model compared to 0.027 and 0.059 for LR + RE model. Additionally, no negative utilities were obtained, so no health state is predicted as being worse than death for both models. Table 2 also shows potential differences between the two models. For example, the predicted mean utility value for the pits state is 0.346 for LR + RE model compared to 0.331 for BR + RE model, whereas the actual mean for this state is 0.322. Further, Table 2 shows the posterior standard deviations of the estimates are mostly bigger for LR + RE model than those for BR + RE model.

**Figure 1:**
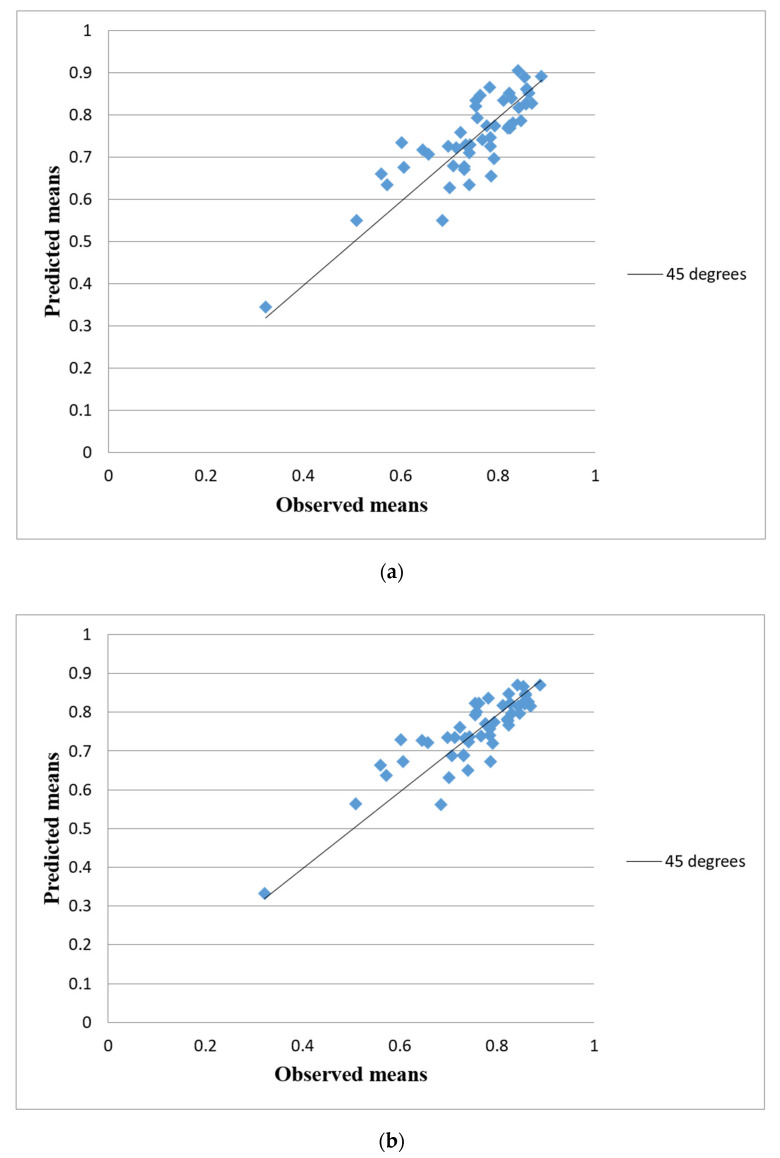
Actual and predicted mean health states valuations for the (**a**) LR + RE model and (**b**) BR + RE model.

Another aspect to show the difference between LR + RE and BR + RE models is presented in Figure 1, which displays, respectively the predicted utilities from the LR + RE model (Figure 1a) and from the BR + RE model (Figure 1b) versus the actual utilities of the 49 health states, along with perfection prediction indicated by a 45° line of unity (solid line). Theoretically, the predicted mean utilities from both models are anticipated to lie roughly on the perfect line; that could be treated as a good agreement. In practice, the plots suggest that the valuations from BR + RE model tend to be closer to the unity line, whereas the valuations from LR + RE model have a larger scatter, so estimates depart largely from the perfect line. This implies that the BR + RE model generates better predictions and much more precise estimates than LR + RE model.

Other spotted performance differences between LR + RE and BR + RE models include the logical monotonicity property that utility should decrease as health decreases in any dimension. Of the total 18,000 SF-6D health states, 10,000 states were selected at random without replacement. Theoretically, each state of the 10,000 has 6–12 states adjacent to it (in a sense, they only differ in one dimension). Then, as a result of selecting one health state at random from these 6–12 states, 10,000 adjacent pairs were obtained. Both models have problems with the non-monotonicity of the predicted mean values for some of these states. However, the extent of non-monotonicity was found to be less for the BR + RE model than the LR + RE one. More specifically, out of these 10,000 adjacent pairs, 15% display non-monotonicity in the LR + RE model versus 10% for the BR + RE model.

**Figure 2:**
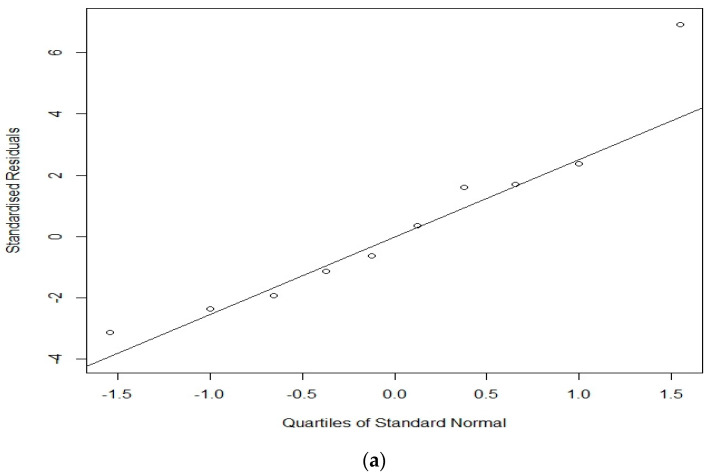

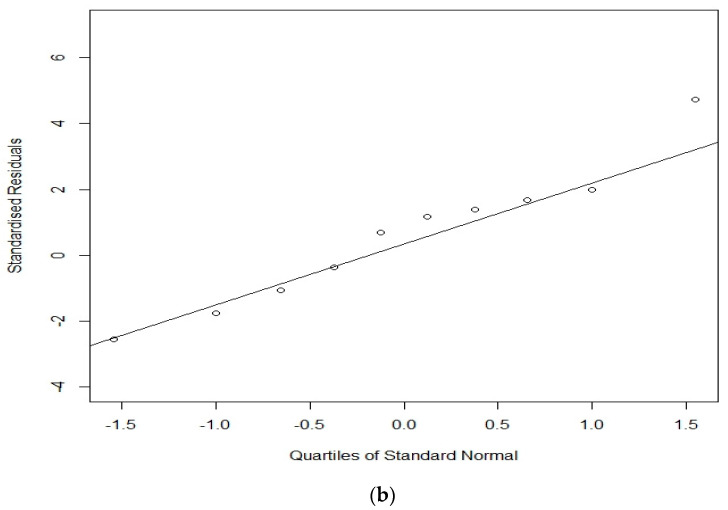
Q–Q plot of standardized predictive errors for the 10 out of sample health states for the (**a**) LR + RE model and (**b**) BR + RE model.

A better test of the validity of the model is to investigate its ability to predict the values for states that have not been used in the estimation. Toward that end, we conduct an out-of-sample leave-one-out prediction, where ten health states were randomly selected from the estimated data; then consecutively, valuations relating to every selected health state were left out, and the two models were implemented on the reduced set of 48 states. Table 3 presents the observed means for the 10 left-out states, along with their predicted means and standard errors from the LR + RE and BR + RE models estimated on the reduced data. Results revealed that the BR + RE model predicts the left-out data quite well and better than LR + RE overall, with RMSE values of 0.091 and 0.107, respectively. Figure 2a,b presents the Q–Q plots of standardized predictive errors for the 10 out of sample health states for LR + RE and BR + RE models, respectively. The solid line in every case represents the theoretical normal distribution. Theoretically, the quantiles of these standardized predictive errors are anticipated to lie roughly on the solid line; hence their distributions are identical. The plots suggest that the quantiles from BR + RE model tend to be closer to the theoretical line, whereas in contrast, the quantiles from LR + RE model have a larger scatter, so estimates deviate largely from the theoretical line. Almost identical results were also obtained across five replicates.

## 4. Discussion

Typically, the distribution of health state values obtained from valuation techniques such as TTO and SG is skewed, truncated, hierarchal and noncontinuous, all of which impose a key challenge for statistical modeling methods. The present study considered the Bayesian framework for beta regression based on three different approaches in order to address the aforementioned characteristics of the HRQoL data. More specifically, this study examined if Bayesian beta regression is appropriate for analyzing the SF-6D health state utility scores and respondent characteristics in a population-based Lebanese health study. For the three different approaches, the beta regression model was found to perform better than the normal regression model under all criteria used.

Further, the beta regression with random effects model performed best, with MPE (0.084), RMSE (0.058) and DIC (−1621), given including covariates did not provide any further improvement in terms of MPE, RMSE and DIC, let alone the 95% CIs for all covariates coefficients contained zero. It is clear that from the analysis presented here that the predictive ability of the beta regression model is better than the previously used normal regression model overall, as reflected in better RMSE within the entire estimation data and better predictive errors in the out-of-sample validation data.

The use of the SF-6D has become widespread across the globe, reaching the US, UK, Europe and Asia, and is largely available for use in datasets since it is derived from the commonly used SF-36. There is potential to borrow strength from existing countries’ valuations to generate better representative utility estimates [33,34,35]. The beta regression model provides a key potential advantage as it allows making use of findings of one country as informative priors to improve those of another, and as such, generated utility estimates of the second country would be much more accurate than collecting and analyzing its data separately. This is a promising approach for other countries with smaller population sizes or low- and middle-income countries (LMIC) where the cost of undertaking large valuation surveys and collecting data via face-to-face interviews with techniques such as SG and/or TTO to develop country-specific value sets could be restrictive. Further research is underway to explore whether using the already existing UK value sets may contribute substantial informative prior to the analysis of the new valuation SF-6D study in Lebanon, and preliminary results sound very promising.

It is to be noted that for the analysis presented here, we have used the SF-6D version 1 (SF-6Dv1). A new revised version of the descriptive system was derived, the SF-6Dv2 [36,37]. This descriptive system still contains the same six dimensions (physical functioning, role functioning, vitality, mental health, social functioning and pain), but these differ from the original SF-6D. The new descriptive system was developed using a recently developed methodology involving a combination of Rasch analysis with classical psychometric analysis. The severity levels of each dimension were selected and worded to ensure there is no ambiguity in the severity levels, and all dimensions are positively worded. Toward this end, it would be interesting to investigate whether or not different results would be obtained when the new instrument (SF-6Dv2) is applied.

The models show that the coefficients associated with pain and mental health are smaller than those for physical functioning or social functioning. These results are different from other studies, including the SF-6Dv1 [6], SF-6Dv2 in the general population [36], SF-6Dv2 in a specific population of patients [38], however, similar to others [39,40]. In a systematic review conducted by Poder and Gandji [41], it is noted that countries populated by native English-speaking persons (UK, USA, Canada and Australia) give more importance to pain than countries outside this cultural world (e.g., Latin and Asian countries). Kharroubi [24,42] argues in this sense too. This motivates the importance of developing a value set for each country as using other countries’ utility values carries some risks in not representing the views and preferences of the population in question, given differences in the sociodemographic and health profile and cultures of the countries [43,44,45].

Limitations of this study include the use of a small sample of 126 people, so this may limit the generalizability of the preference values found. The small number of health states valued could impact the accuracy of econometric modeling. Therefore, the generated SF-6D preference-based coefficients from this pilot study should not be regarded as necessarily representative of the general population of Lebanon. An additional limitation is that all the models outlined above used “vague” prior distributions, which may produce little influence on the overall findings. In a fully Bayesian framework, including external information within the prior distribution would be eminently possible. Such information would be desirable to study the impact that future predicted healthcare reforms may have on HRQoL, and hence allow for more pertinent prediction of future utilities. Another limitation is that we did not explore the predictive ability of other complex regression models. Other investigators have explored generalized linear models, TPM and survival-type models, to estimate HRQoL outcomes [46,47]. Although these models had greater predictive ability compared with linear regression in these studies, it is apparent from the literature that the ideal model is heavily dependent on the derivation data. Linear regression models are the most widely used type of predictive models and, despite their theoretical limitations, appear to be robust in practice. All of the above are the subject of further research.

The standard gamble technique was chosen as the main method for eliciting preferences since it has a strong theoretical foundation in expected utility theory, asking respondents to trade changes in health against risk. It is to be noted that gamble theory has been applied extensively in the health literature on issues such as whether parents act as a risk or protective factor for their children’s alcohol consumption [48,49], sexually transmitted infections [50] or even fear of missing out [51]. Future research is then recommended to explore whether physical functioning, role limitations, social functioning, pain, mental health, and vitality would act as risk or protector factor in Lebanese society. It would also be interesting to investigate whether this perspective would be different with beta and linear regression models and to know whether the risk factors detected with one regression model are problematic factors when another model is applied [52].

The practical importance of the differences in average health state values between the beta and linear regression models depends on their impact on the relative cost per QALY of different treatments. The impact on cost per QALY relies on the nature of the efficacy (e.g., with it having more of an impact for survival differences), the severity of the patients’ state of health (e.g., the relative importance of the differences is going to be higher for patients with lower utility scores), cost and how close the incremental cost per QALY of treatment is to the willingness-to-pay threshold of the reimbursement agency. The consequences of the models presented should be further explored for patient groups [53].

The beta regression Bayesian model presented here provides a flexible approach to modeling health state values for different instruments like EQ-5D and HUI-II, in addition to more specialized, condition-specific measures. It would then be of interest to determine and compare the minimally important difference for the different measures and conditions for various datasets [53,54]. Further, such a model provides important information for the planning of future services and budgets and could also be implemented to inform CEA, in particular, assessing the effectiveness of healthcare spending among the LMIC/Asian economies [55,56].

## 5. Conclusions

In conclusion, this present paper considered the Bayesian framework for beta regression based on three different approaches for analyzing the SF-6D health state utility scores and respondent characteristics in a population-based Lebanese health study. For the three different approaches, the beta regression model was found to perform better than the normal regression model under all criteria used. Overall, the beta regression with random effects model performed best and, compared to the traditionally used linear regression model, provided better predictions of observed values in the entire learning sample and in an out-of-sample validation. Such a model will be of significant use to investigators modeling and predicting utility weights for use in economic evaluations. We hope that this work will provide applied researchers with a practical set of tools to appropriately model outcomes in CEA.

## Figures and Tables

**Table 1:** Model coefficients and model performance (95% credible interval in parentheses).

Parameter	LR	BR	LR + RE	BR + RE	LR + RE + COV	BR + RE +COV
**β**	0.964 (0.916, 1.012)	2.024 (1.795, 2.254)	0.938 (0.894, 0.981)	2.175 (1.941, 2.443)	0.857 (0.726, 0.998)	1.666 (1.045, 2.443)
**β PF2**	**−0.043 (−0.079, −0.006)**	**−0.199 (−0.371, −0.028)**	**−0.047 (−0.072, −0.022)**	**−0.255 (−0.410, −0.096)**	**−0.046 (−0.072, −0.021)**	**−0.252 (−0.406, −0.098)**
**β PF3**	**−0.044 (−0.081, −0.007)**	**−0.204 (−0.379, −0.025)**	**−0.046 (−0.072, −0.021)**	**−0.243 (−0.402, −0.083)**	**−0.046 (−0.071, −0.020)**	**−0.238 (−0.395, −0.080)**
**β PF4**	**−0.057 (−0.094, −0.020)**	**−0.276 (−0.447, −0.101)**	**−0.059 (−0.084, −0.033)**	**−0.343 (−0.497, −0.187)**	**−0.059 (−0.084, −0.033)**	**−0.343 (−0.502, −0.190)**
**β PF5**	**−0.094 (−0.131, −0.057)**	**−0.408 (−0.580, −0.236)**	**−0.096 (−0.122, −0.071)**	**−0.515 (−0.667, −0.363)**	**−0.095 (−0.121, −0.069)**	**−0.511 (−0.663, −0.359)**
**β PF6**	**−0.171 (−0.206, −0.137)**	**−0.740 (−0.898, −0.582)**	**−0.165 (−0.189, −0.141)**	**−0.797 (−0.940, −0.659)**	**−0.165 (−0.189, −0.141)**	**−0.796 (−0.934, −0.660)**
**β RL2**	**−0.041 (−0.070, −0.011)**	**−0.190 (−0.326, −0.053)**	**−0.042 (−0.075, −0.009)**	**−0.194 (−0.377, −0.006)**	**−0.042 (−0.075, −0.009)**	**−0.183 (−0.374, 0.007)**
**β RL3**	0.005 (−0.025, 0.035)	0.002 (−0.136, 0.140)	−0.024 (−0.054, 0.006)	−0.118 (−0.294, 0.054)	−0.024 (−0.054, 0.006)	−0.11 (−0.277, 0.063)
**β RL4**	**−0.056 (−0.090, −0.022)**	**−0.227 (−0.382, −0.072)**	**−0.109 (−0.141, −0.077)**	**−0.488 (−0.673, −0.303)**	**−0.108 (−0.141, −0.076)**	**−0.475 (−0.657, −0.298)**
**β SF2**	**−0.051 (−0.081, −0.021)**	**−0.216 (−0.360, −0.072)**	**−0.040 (−0.062, −0.019)**	**−0.224 (−0.361, −0.086)**	**−0.040 (−0.062, −0.019)**	**−0.223 (−0.359, −0.089)**
**β SF3**	**−0.094 (−0.131, −0.058)**	**−0.39 (−0.563, −0.216)**	**−0.037 (−0.066, −0.009)**	**−0.224 (−0.397, −0.052)**	**−0.037 (−0.066, −0.008)**	**−0.22 (−0.397, −0.045)**
**β SF4**	**−0.110 (−0.146, −0.073)**	**−0.468 (−0.638, −0.296)**	**−0.058 (−0.088, −0.028)**	**−0.333 (−0.519, −0.153)**	**−0.058 (−0.088, −0.028)**	**−0.331 (−0.514, −0.151)**
**β SF5**	**−0.169 (−0.203, −0.135)**	**−0.724 (−0.883, −0.570)**	**−0.105 (−0.133, −0.076)**	**−0.529 (−0.693, −0.369)**	**−0.105 (−0.133, −0.077)**	**−0.530 (−0.692, −0.363)**
**β PAIN2**	−0.021 (−0.058, 0.015)	−0.102 (−0.272, 0.072)	−0.003 (−0.029, 0.023)	−0.074 (−0.231, 0.081)	−0.003 (−0.030, 0.023)	−0.078 (−0.234, 0.083)
**β PAIN3**	−0.006 (−0.043, 0.030)	−0.042 (−0.214, 0.129)	0.014 (−0.012, 0.040)	0.002 (−0.156, 0.160)	0.013 (−0.013, 0.039)	−0.006 (−0.164, 0.157)
**β PAIN4**	−0.020 (−0.057, 0.016)	−0.091 (−0.263, 0.085)	−0.011 (−0.038, 0.016)	−0.088 (−0.252, 0.073)	−0.011 (−0.038, 0.016)	−0.091 (−0.256, 0.078)
**β PAIN5**	**−0.052 (−0.089, −0.016)**	**−0.228 (−0.397, −0.057)**	−0.010 (−0.039, 0.017)	−0.132 (−0.296, 0.031)	−0.011 (−0.039, 0.018)	−0.139 (−0.303, 0.024)
**β PAIN6**	**−0.107 (−0.142, −0.072)**	**−0.481 (−0.634, −0.326)**	**−0.086 (−0.110, −0.061)**	**−0.428 (−0.572, −0.286)**	**−0.086 (−0.111, −0.062)**	**−0.434 (−0.578, −0.293)**
**β MH2**	−0.001 (−0.030, 0.029)	0.005 (−0.134, 0.148)	−0.001 (−0.023, 0.022)	−0.027 (−0.161, 0.108)	−0.001 (−0.024, 0.022)	−0.028 (−0.163, 0.107)
**β MH3**	−0.014 (−0.051, 0.022)	−0.086 (−0.253, 0.087)	−0.026 (−0.051, −0.000)	−0.152 (−0.308, 0.005)	−0.026 (−0.052, −0.000)	−0.153 (−0.308, 0.001)
**β MH4**	**−0.088 (−0.124, −0.052)**	**−0.388 (−0.558, −0.220)**	**−0.075 (−0.101, −0.048)**	**−0.392 (−0.548, −0.239)**	**−0.075 (−0.102, −0.049)**	**−0.395 (−0.547, −0.241)**
**β MH5**	**−0.069 (−0.104, −0.035)**	**−0.341 (−0.491, −0.187)**	**−0.076 (−0.102, −0.051)**	**−0.392 (−0.539, −0.248)**	**−0.077 (−0.103, −0.051)**	**−0.397 (−0.544, −0.250)**
**β VIT2**	0.001 (−0.029, 0.030)	0.020 (−0.119, 0.16)	0.011 (−0.011, 0.033)	0.016 (−0.113, 0.144)	0.012 (−0.010, 0.033)	0.023 (−0.109, 0.154)
**β VIT3**	0.016 (−0.021, 0.052)	0.091 (−0.084, 0.264)	−0.001 (−0.027, 0.026)	−0.037 (−0.199, 0.125)	0.000 (−0.027, 0.027)	−0.030 (−0.191, 0.137)
**β VIT4**	−0.034 (−0.070, 0.002)	−0.131 (−0.300, 0.040)	−0.017 (−0.043, 0.009)	−0.126 (−0.276, 0.028)	−0.017 (−0.043, 0.009)	−0.127 (−0.284, 0.028)
**β VIT5**	**−0.037 (−0.072, −0.003)**	**−0.152 (−0.304, 0.002)**	**−0.051 (−0.077, −0.026)**	**−0.246 (−0.391, −0.102)**	**−0.050 (−0.076, −0.024)**	**−0.240 (−0.391, −0.089)**
**β AGE**	NA	NA	NA	NA	0.001 (−0.002, 0.003)	0.006 (−0.007, 0.019)
**β GENDER**	NA	NA	NA	NA	−0.025 (−0.072, 0.022)	−0.096 (−0.317, 0.157)
**β DEGREE**	NA	NA	NA	NA	0.060 (−0.022, 0.139)	0.298 (−0.025, 0.633)
**β HOUSING1**	NA	NA	NA	NA	−0.029 (−0.097, 0.041)	−0.083 (−0.426, 0.252)
**β HOUSING2**	NA	NA	NA	NA	−0.014 (−0.089, 0.060)	−0.006 (−0.382, 0.355)
**β INCOME1**	NA	NA	NA	NA	0.003 (−0.086, 0.090)	−0.013 (−0.417, 0.376)
**β INCOME2**	NA	NA	NA	NA	0.002 (−0.066, 0.066)	−0.026 (−0.317, 0.243)
**β MS1**	NA	NA	NA	NA	0.067 (−0.004, 0.141)	0.358 (−0.006, 0.715)
**β MS2**	NA	NA	NA	NA	0.069 (−0.126, 0.270)	0.380 (−0.527, 1.338)
**σ**	NA	NA	NA	NA	NA	NA
*ϕ*	NA	**6.924 (6.334, 7.531)**	NA	**9.943 (9.793, 9.999)**	NA	**9.944 (9.794, 9.999)**
**MPE**	0.128	0.126	0.089	0.084	0.089	0.084
**RMSE**	0.053	0.049	0.064	0.058	0.116	0.113
**DIC**	−689.1	−1069	−1325	−1621	−1306	−1605

**Note: LR**: linear regression; **BR**: beta regression; **RE**: random effect; **COV**: covariates; **CI**: credible interval; **PF**, physical functioning; **RL**, role limitations; **SF**, social functioning; **PAIN**, pain; **MH**, mental health; **VIT**, vitality; **NA**, not applicable; **MPE**: mean prediction error; **RMSE**: root mean square error; **DIC**: deviance information criterion. Values given as posterior mean (95% credible interval). The number next to each parameter (2, 3, 4, 5, and 6) refers to the level within each dimension. Parameters estimates highlighted in bold are those who have credible intervals excluding zero.

**Table 2:** Inference for the 49 health states.

		Predicted			
Health	Observed	LR + RE		BR + RE	
State	Mean	Mean	SD	Mean	SD
111,621	0.824	0.852	0.023	0.847	0.017
113,411	0.854	0.890	0.023	0.865	0.016
115,653	0.730	0.671	0.024	0.687	0.028
121,212	0.842	0.904	0.023	0.872	0.014
122,233	0.869	0.826	0.024	0.816	0.021
122,425	0.758	0.793	0.023	0.801	0.021
124,125	0.848	0.786	0.023	0.797	0.021
131,542	0.828	0.841	0.022	0.824	0.019
132,524	0.763	0.846	0.023	0.824	0.019
133,132	0.858	0.862	0.022	0.844	0.017
135,312	0.756	0.835	0.024	0.824	0.019
142,154	0.791	0.696	0.024	0.719	0.027
144,341	0.742	0.711	0.026	0.723	0.028
211,111	0.890	0.891	0.021	0.872	0.014
212,145	0.785	0.725	0.025	0.742	0.027
213,323	0.783	0.866	0.026	0.836	0.021
221,452	0.824	0.773	0.025	0.778	0.024
224,612	0.646	0.717	0.026	0.726	0.029
232,111	0.858	0.827	0.022	0.828	0.018
235,224	0.767	0.741	0.026	0.739	0.028
241,531	0.785	0.746	0.026	0.758	0.028
312,332	0.864	0.851	0.025	0.828	0.021
315,515	0.698	0.726	0.027	0.735	0.029
321,122	0.858	0.861	0.022	0.848	0.016
323,644	0.571	0.635	0.029	0.638	0.036
332,411	0.844	0.817	0.024	0.817	0.021
334,251	0.734	0.730	0.027	0.733	0.030
341,123	0.831	0.782	0.027	0.798	0.025
412,152	0.793	0.774	0.024	0.773	0.023
414,522	0.755	0.821	0.028	0.794	0.026
421,314	0.811	0.835	0.025	0.819	0.022
425,131	0.658	0.707	0.026	0.722	0.029
431,443	0.824	0.770	0.027	0.767	0.027
432,621	0.743	0.729	0.024	0.737	0.026
443,215	0.731	0.679	0.027	0.689	0.032
511,114	0.858	0.825	0.024	0.822	0.020
512,242	0.603	0.735	0.025	0.728	0.028
522,321	0.777	0.773	0.023	0.771	0.022
523,551	0.607	0.676	0.027	0.671	0.032
531,635	0.786	0.656	0.026	0.671	0.031
534,113	0.723	0.759	0.025	0.763	0.026
545,422	0.700	0.628	0.026	0.632	0.032
611,221	0.821	0.769	0.024	0.781	0.023
614,434	0.561	0.661	0.028	0.663	0.034
622,513	0.707	0.680	0.024	0.687	0.029
625,141	0.510	0.552	0.026	0.565	0.034
631,355	0.741	0.636	0.025	0.650	0.031
633,122	0.714	0.722	0.023	0.735	0.025
642,612	0.685	0.550	0.022	0.563	0.028
645,655	0.322	0.346	0.015	0.331	0.017
MPE		0.032		0.027	
RMSE		0.059		0.053	

Note: LR: linear regression; BR: beta regression; RE: random effect; MPE: mean prediction error; RMSE: root mean square error; SD: standard deviation.

**Table 3:** Out of sample predictions for 10 health states.

Omitted Health State	Observed Mean		LR + RE			BR + RE	
		Mean	SD	SR	Mean	SD	SR
121,212	0.842	0.876	0.032	−1.129	0.849	0.024	−0.368
132,524	0.763	0.841	0.034	−2.367	0.830	0.028	−2.555
211,111	0.890	0.909	0.029	−0.637	0.866	0.021	1.172
232,111	0.858	0.805	0.033	1.705	0.798	0.031	1.983
321,122	0.858	0.848	0.032	0.364	0.842	0.026	0.694
412,152	0.793	0.733	0.035	1.606	0.724	0.040	1.666
432,621	0.743	0.647	0.039	2.385	0.664	0.054	1.388
523,551	0.670	0.701	0.048	−1.920	0.677	0.062	−1.072
614,434	0.561	0.742	0.058	−3.123	0.669	0.062	−1.756
642,612	0.685	0.458	0.032	6.924	0.462	0.046	4.728
RMSE		0.107			0.091		

Note: LR: linear regression; BR: beta regression; RE: random effect; RMSE: root mean square error; SD: standard deviation; SR: standardized residuals.
